# Chemical coding and chemosensory properties of cholinergic brush cells in the mouse gastrointestinal and biliary tract

**DOI:** 10.3389/fphys.2015.00087

**Published:** 2015-03-24

**Authors:** Burkhard Schütz, Innokentij Jurastow, Sandra Bader, Cornelia Ringer, Jakob von Engelhardt, Vladimir Chubanov, Thomas Gudermann, Martin Diener, Wolfgang Kummer, Gabriela Krasteva-Christ, Eberhard Weihe

**Affiliations:** ^1^Department of Molecular Neuroscience, Institute of Anatomy and Cell Biology, Philipps-UniversityMarburg, Germany; ^2^Institute of Anatomy and Cell Biology, Justus-Liebig-UniversityGiessen, Germany; ^3^Institute of Veterinary Physiology and Biochemistry, Justus-Liebig-UniversityGiessen, Germany; ^4^Synaptic Signaling and Neurodegeneration, German Center for Neurodegenerative Diseases (DZNE)Bonn, Germany; ^5^German Cancer Research Center (DKFZ)Heidelberg, Germany; ^6^Walther-Straub-Institute for Pharmacology and Toxicology, Ludwig-Maximilians-UniversityMunich, Germany; ^7^Institute of Anatomy and Cell Biology, Julius-Maximilians-UniversityWürzburg, Germany

**Keywords:** brush cell, cholinergic, ChAT, VAChT, ChT1, intestine, gall bladder, bile duct

## Abstract

The mouse gastro-intestinal and biliary tract mucosal epithelia harbor choline acetyltransferase (ChAT)-positive brush cells with taste cell-like traits. With the aid of two transgenic mouse lines that express green fluorescent protein (EGFP) under the control of the ChAT promoter (EGFP^*ChAT*^) and by using *in situ* hybridization and immunohistochemistry we found that EGFP^*ChAT*^ cells were clustered in the epithelium lining the gastric groove. EGFP^*ChAT*^ cells were numerous in the gall bladder and bile duct, and found scattered as solitary cells along the small and large intestine. While all EGFP^*ChAT*^ cells were also ChAT-positive, expression of the high-affinity choline transporter (ChT1) was never detected. Except for the proximal colon, EGFP^*ChAT*^ cells also lacked detectable expression of the vesicular acetylcholine transporter (VAChT). EGFP^*ChAT*^ cells were found to be separate from enteroendocrine cells, however they were all immunoreactive for cytokeratin 18 (CK18), transient receptor potential melastatin-like subtype 5 channel (TRPM5), and for cyclooxygenases 1 (COX1) and 2 (COX2). The *ex vivo* stimulation of colonic EGFP^*ChAT*^ cells with the bitter substance denatonium resulted in a strong increase in intracellular calcium, while in other epithelial cells such an increase was significantly weaker and also timely delayed. Subsequent stimulation with cycloheximide was ineffective in both cell populations. Given their chemical coding and chemosensory properties, EGFP^*ChAT*^ brush cells thus may have integrative functions and participate in induction of protective reflexes and inflammatory events by utilizing ACh and prostaglandins for paracrine signaling.

## Introduction

Besides conventional absorptive enterocytes, the mucosal epithelium of the gastro-intestinal (GI) tract harbors many specialized chemosensory cells that detect nutrients and/or poisons and regulate GI functions. Many of these cells are open or closed-type enteroendocrine cells and produce a single or combinations of peptide hormones and secrete them in a vesicle-operated manner (Brubaker, 2012). While in the oral cavity the composition of nutrients is registered by taste cells in lingual taste buds, an additional possibly taste perceiving-like cell type distinct from enteroendocrine cells was identified in the rodent GI tract. Based on its morphological appearance with a tuft of stiff microvilli at its apex this cell type was referred to as “tuft cell” (Sato, 2007) or “brush cell” (Trier et al., 1987; Höfer and Drenckhahn, 1992). GI brush cells represent about 0.4% of the epithelial cells and express membrane-bound receptors and intracellular signal transduction molecules that, in this combination, have been attributed only to certain taste-perceiving cells in taste buds of the oral cavity (Iwatsuki and Uneyama, 2012). This includes receptors for bitter (Wu et al., 2002), sweet (Dyer et al., 2005), and umami (Bezençon et al., 2007) taste, the G-protein α-gustducin (Höfer et al., 1996), phospholipase β2 (PLCβ2, Rössler et al., 1998,) and TRPM5 (Pérez et al., 2002; Hofmann et al., 2003; Kaske et al., 2007). In mice, a cluster of brush cells was identified in the mucosal lining of the gastric groove (Hass et al., 2007), while in the intestine (Bezençon et al., 2007), gall bladder and bile/pancreatic ducts (Luciano and Reale, 1997; Höfer and Drenckhahn, 1998) they are found scattered as solitary entities.

Taste cells in taste buds communicate with innervating nerve fibers via release of ATP as neurotransmitter (Finger et al., 2005; Chaudhari and Roper, 2010). Because some taste cells express ChAT, the enzyme that catalyzes the synthesis of ACh from its precursor choline and acetyl-CoA (Ogura et al., 2007), this classical neurotransmitter has been proposed as an additional signaling substance. The occurrence of solitary ChAT immunoreactive cells in the mucosal epithelium of the human large intestine has been reported about two decades ago (Porter et al., 1996), and the presence and enzymatic activity of ChAT was further validated in surface cells from rat and human intestine (Klapproth et al., 1997). Based on the presence of ChAT, the list of non-neuronal ACh-producing cells and tissues is growing (Wessler et al., 1998). It was not until recently, however, that ChAT expression along the gastro-intestinal tract has been unequivocally attributed to the proposed chemosensory brush cells. ChAT was found to be present in PLCβ2-positive brush cells in the gastric groove epithelium using *in situ* hybridization and immunohistochemistry (Eberle et al., 2013b), and in the small intestine via the use of transgenic expression from the ChAT gene locus of the fluorescent proteins enhanced green fluorescent protein (EGFP) (Tallini et al., 2006) or tdTomato (Gautron et al., 2013). Yet it is still unresolved if gastro-intestinal brush cells, comparable to non-neuronal cholinergic brush cells in the airways (Krasteva et al., 2011), express the full “neuronal-type” complement of ACh handling proteins, i.e., ChAT, VAChT, required for the uptake of ACh into small synaptic vesicles (Erickson et al., 1994), and the high-affinity choline transporter (ChT1), required for the re-uptake of choline to fuel intracellular ACh synthesis (Okuda and Haga, 2000; Ogura et al., 2007). At least in human non-neuronal cholinergic cells the expression of the cholinergic gene locus (CGL) (Eiden, 1998) seems to be incomplete as vesicular acetylcholine transporter (VAChT) expression has never been found in non-neuronal sites of the human gut by using *in situ* hybridization (Anlauf et al., 2003), indicating major differences in ACh synthesis, release and recycling between neuronal and non-neuronal cholinergic cells (Kummer et al., 2008). Even more challenging, the types of stimuli that elicit responses in gastro-intestinal cholinergic brush cells are still enigmatic.

To investigate if the non-neuronal presumed cholinergic brush cells in the mouse GI and biliary tracts also have a neuron typical cholinergic phenotype, we took advantage of two independently generated transgenic mouse lines that express EGFP under the control of the ChAT promoter (Tallini et al., 2006; von Engelhardt et al., 2007). These two mouse lines have already successfully been used to visualize cholinergic neurons in the central and peripheral nervous systems (Schütz et al., 2008), cholinergic taste cells in lingual taste buds (Ogura et al., 2007), and solitary chemosensory cells in trachea (Krasteva et al., 2011), auditory tube (Krasteva et al., 2012b), and urethra (Deckmann et al., 2014). Here, we have performed a detailed molecular expression profile analysis of EGFP^*ChAT*^-expressing non-neuronal cholinergic brush cells, and functional characterization of the responsiveness of these cells to bitter stimuli *ex vivo* in colon tissue preparations and in isolated cells.

## Materials and methods

### Animal strains

Two lines of BAC-transgenic mice that express EGFP under the control of the ChAT promoter were used (Tallini et al., 2006; von Engelhardt et al., 2007). Mice were housed in groups of 3–6 in single ventilated cages under specified pathogen-free conditions. They were kept on a 12 h light/12 h dark cycle and had access to food and water *ad libitum*. All animal procedures were conducted in accordance with EU Directive 2010/63/EU for animal experiments, the German Animal Protection Law, and protocols approved by the county administrative government in Gießen (A8/2010), or by the local veterinarian (Ex-8-2013). Hemizygous transgenic mice were mated with wild type C57BL/6N mice to obtain litters for experimental analysis. Successful transmission of the transgene was verified by PCR for the encoded EGFP using genomic DNA obtained from ear biopsies (von Engelhardt et al., 2007; Schütz et al., 2008). Mice of both sexes were 8–20 weeks of age when used. Both mouse strains showed identical EGFP expression patterns, therefore all data presented in the following sections are gathered from one of these lines (von Engelhardt et al., 2007).

### Tissue harvesting for histological analysis

For cellular and molecular analyses, a total of eight transgenic (four male and four female) and three non-transgenic (male) mice, all 11–15 weeks of age, were sedated by inhalation of isoflurane, and sacrificed by cervical dislocation. For the visualization of native EGFP fluorescence, the whole gall bladder was dissected, cut open, mounted flat on a microscopic slide, and cover-slipped. For *in situ* hybridization and immunohistochemistry, the whole stomach was removed, opened along the large curvature, and the content washed out. The whole gall bladder was left attached to pieces of surrounding liver tissue. Also, 2–4 pieces of tissue each from duodenum (including pancreas), jejunum, ileum and colon, all 0.5–1 cm in length, were quickly dissected. The tissue was either directly frozen in −40°C cold isopentane, or immersion-fixed in Bouin Hollande fixative (Schütz et al., 2008). For further analysis all tissues were flat-mounted to obtain longitudinal profiles during sectioning.

### *In situ* hybridization

Serial 14 μm thick sections were cut with a cryostat and mounted on silanized glass slides. Complementary RNA probes for the detection of mouse ChAT and ChT1 transcripts in tissue sections were generated from mouse C57BL/6 brainstem cDNA. For ChAT, a 758 nt long DNA fragment (GeneBank acc. no. NM_003891), and for ChT1 a 836 nt long DNA fragment (GeneBank acc. no. NM_022025) were amplified by PCR, subcloned into pGEM-T (Promega, Mannheim, Germany), and the sequence confirmed by double-stranded sequencing. For the detection of EGFP transcripts, a 601 bp fragment from the EGFP coding sequence (pEGFP-N1, Clontech, Palo Alto, USA) was amplified by PCR using primers TGT AAT ACG ACT CAC TAT AGG GGA CGT AAA CGG CCA CAA GTT C (with 5′ SP6 site) and TGA TTT AGG TGA CAC TAT AGA AGC AGG ACC ATG TGA TCG C (with 5′ T7 site). The generation of complementary RNA probes for the detection of mouse VAChT transcripts in tissue sections has been described previously (Schütz et al., 2008). Gene-specific radioactively (35-S)-labeled transcripts were generated using SP6 (for antisense probe) and T7 (for sense probe) RNA polymerases. The *in situ* hybridization histochemistry procedure was essentially performed as described in detail earlier (Schäfer et al., 1997; Schütz et al., 2000). Briefly, tissue sections on microscopic slides were covered with 30–40 μl of hybridization solution, containing 50% formamide, 0.6 M NaCl, 10 mM Tris (pH 7.4), 1 mM Na_2_EDTA, 1 X Denhardts, 10% dextran sulfate, 100 mg/ml sheared salmon sperm DNA, 0.05% (w/v) *E. coli* MRE600 tRNA, 20 mM dithiothreitol (DTT), 50,000 d.p.m./ml riboprobe, and coverslipped. Hybridization was carried out for 16 h at 60°C in a humid chamber. After hybridization, coverslips were removed in 2 × standard sodium citrate (SSC) at room temperature and the sections washed in the following order: 20 min in 1 × SSC, 45 min at 37°C in RNase solution (20 mg/ml RNase A and 1 U/ml RNase T1), 20 min in 1 × SSC, 20 min in 0.5 × SSC, 20 min in 0.2 × SSC, 60 min in 0.2 × SSC at 60°C, 10 min in 0.2 × SSC at room temperature and finally 10 min in distilled water. The sections were dehydrated in 50 and 70% ethanol and air dried at room temperature. For visualization of hybridization signals, sections were first exposed to Kodak BioMax MR autoradiography film (Sigma-Aldrich, Steinheim, Germany) for 3 days to estimate further exposure times, then coated under absence of light with Kodak NTB autoradiography emulsion (Eastman Kodak, Rochester, NY, USA), exposed for 1–4 weeks at 4°C in the dark and finally developed. Sections were counterstained with cresyl violet, dehydrated and coverslipped. Images were taken as specified below.

### Immunohistochemistry

All immunohistochemical procedures were performed essentially as described earlier (Schütz et al., 2008). Briefly, paraffin-embedded sections mounted on microscopic slides were deparaffinized with xylene, rehydrated through a graded series of 2-propanol, incubated in methanol/hydrogen peroxide (0.3% v/v) to block endogenous peroxidase activity and then incubated in 10 mM sodium citrate buffer (pH 6.0) at 95°C for 10 min for antigen retrieval. Unspecific antibody binding was blocked by incubation in 5% bovine serum albumin (BSA)/50 mM PBS (pH 7.4) for 1 h. For single-labeling brightfield immunohistochemistry, primary antibodies were applied in 1% BSA/PBS and incubated at 16°C overnight followed by 2 h at 37°C. Details about the primary and secondary antibodies used are given in Table 1. Negative control staining was performed by omission of primary antibody. After 3 × 10 min wash in PBS, sections were incubated with species-specific biotinylated secondary antibodies (Dianova, Hamburg, Germany; diluted 1:500 in 1%BSA/PBS, see Table 1) for 45 min at 37°C, and the antigen-antibody complexes visualized with the Vectastain Elite ABC kit (Vector Laboratories, Burlingame, CA, USA), using diaminobenzidine/nickel as substrate. In case of ChT1 detection in duodenum and colon, additional tyramide signal amplification was performed using a TSA Kit (NEL700, PerkinElmer, Waltham, MA, USA) following the instructions of the manufacturer. For double-immunofluorescence microscopy, a combination of the two primary antibodies was co-applied in 1% BSA/PBS, as described (Schütz et al., 2008). Incubation was carried out overnight at 16°C, followed by 2 h at 37°C. After extensive washing with 1%BSA/PBS over 30 min, immunoreactions were visualized for the first primary antibody by species-specific secondary antibodies labeled with Alexa Fluor 647 (MoBiTec, Göttingen, Germany; dilution 1:200 in 1%BSA/PBS) and for the second primary antibody by species-specific biotinylated secondary antisera (Dianova; diluted 1:200 in 1%BSA/PBS) followed by streptavidin-conjugated Alexa Fluor 488 (1:200 in 1%BSA/PBS). All secondary antibodies and streptavidin conjugates were applied for 2 h at 37°C. Sections were extensively washed before they were coverslipped with FluorSave reagent (Calbiochem, Merck Biosciences, Schwalbach, Germany). Immunofluorescence staining was documented as digitized false-color images (8-bit Tiff format) obtained with an Olympus BX50WI laser scanning microscope (Olympus Optical, Hamburg, Germany) and Olympus Fluoview 2.1 software. Adobe Photoshop 9.0 CS2 software was used to compose and label the plates from single Tiff images without manipulations of contrast or brightness.

**Table 1:** **Characteristics of primary and secondary antibodies used for immunohistochemistry (in alphabetical order)**.

**Antigen**	**Abbreviations**	**Epitope**	**Host species**	**Dilution (BF/IF)**	**Source**	**Catalog no./(internal code)**
**PRIMARY ANTIBODIES**						
Cholecystokinin	CCK	Cholecystokinin-8	Rabbit	n.a./500	Yanaihara	YP030
Choline acetyltransferase	ChAT	Human ChAT	Goat	1000/100	Chemicon	AB144P
Cholintransporter 1^c^	ChT1	Peptide fragment “CALLDVDSSPEGSGTEDNLQ” (C-20-Q) of rat high-affinity choline transporter	Rabbit	3000/500	L. Eiden, NIH, Bethesda, USA	(R473/2A)
Chromogranin A^a^	CGA	Bovine CGA (aa 316-329, WE-14 epitope)	Rabbit	10,000/1000	L. Eiden, NIH, Bethesda, USA	(Lenny 10)
Cyclooxygenase 1	COX1	Peptide from carboxy terminus of human origin	Goat	n.a./500	Santa Cruz	sc-1752
Cyclooxygenase 2	COX2	Peptide from carboxy terminus of human origin	Goat	n.a./500	Santa Cruz	sc-1745
Cytokeratin 18	CK18	Synthetic peptide corresponding to C-terminus of human cytokeratin 18	Rabbit	n.a./200	Spring Bioscience	SP69
Endorphin β	β End	Synthetic human β End	Rabbit	n.a./200	INC/IBL	20063
Enhanced Green Fluorescent Protein	EGFP	EGFP	Rabbit	10,000/ 1000	Life Tech.	A6455
Enhanced Green Fluorescent Protein	EGFP	Pentamer peptide	Sheep	10,000/1000	Dianova	OSS00005W
Gastric Inhibitory Peptide	GIP	Synthetic human GIP	Rabbit	n.a./5000	Yanaihara	Y101
Neurotensin^a^	NT	Neurotensin	Rabbit	n.a./200	R. Carraway, Worcester, UK	n.a.
Peptide Tyrosine Tyrosine^a^	PYY	Porcine PYY	Rabbit	n.a./4000	Milab	B52-100
Secretin	Secr	Human Secretin	Rabbit	n.a./400	Yanaihara	Y120
Serotonin^a^	Sero	Serotonin	Rabbit	n.a./4000	INC/IBL	60080
Somatostatin^a^	SOM	Somatostatin	Rabbit	n.a./200	Serotec	PEPA38
Substance P	SP	Substance P	Rabbit	n.a./1000	B. Eskay, NIH, Bethesda, USA	n.a.
Transient Receptor Potential Cation Channel, subfamily M, member 5^d^	TRPM5	Peptide “ARDREYLESGLPPSDT,” coupled via the N-terminus to keyhole limpet hemocyanin (AB-321)	Rabbit	n.a./1000	V. Chubanov/T. Gudermann, Munich, Germany	n.a.
Vesicular Acetylcholine Transporter^b^	VAChT	Rat VAChT (80259; 11 aa from C-terminus)	Rabbit	5000/500	L. Eiden, NIH, Bethesda, USA	(80259)
**Antigen**	**Ig size**	**Host species**	**Dilution**	**Conjugate**	**Source**	**Catalog no**.
**SECONDARY ANTIBODIES**						
Rabbit IgG	Whole molecule	Donkey	1:200	Biotin	Dianova	711-065-152
Rabbit IgG	Whole molecule	Chicken	1:200	Alexa647	Life Technologies (Molecular Probes)	A21443
Goat IgG	Whole molecule	Donkey	1:200	Biotin	Dianova	705-065-147
Sheep IgG	Whole molecule	Donkey	1:200	Alexa647	Life Technologies (Molecular Probes)	A21448
n.a.	n.a.	n.a.	1:200	Streptavidin-Alexa488	Life Technologies (Molecular Probes)	S11223

Abbreviations: BF, brightfield; IF, immunofluorescence; n.a., not applicable. Companies, Abcam; Cambridge, UK; Biozol, Eching, Germany; Dianova, Hamburg, Germany; INC/IBL, Minneapolis, USA; Life Technologies, Carlsbad, USA; Milab, Malmö, Sweden; Santa Cruz, Heidelberg, Germany; Serotec, Oxford, UK; Spring Bioscience, Pleasanton, USA; Yanaihara Laboratories, Shizuoka, Japan (

a Schäfer et al., 1994;

b Weihe et al., 1996;

c Schütz et al., 2004;

d Kaske et al., 2007).

### Colon cell isolation

Explanted pieces of colon were enzymatically digested for 30 min at 37°C in 1 ml minimal essential medium (MEM; Invitrogen, Darmstadt, Germany), containing papain (2 mg/ml), BSA (2 mg/ml), DTT (0.5 mg/ml), and 10 μl each of L-cysteine and P/S (penicillin (100 U/ml) and streptomycin (100 μg/ml), centrifuged (600 rpm, 5 min) and mechanically dissociated. Inactivation of papain via leupeptine (2 μl/ml) was followed by a second enzymatic digestion in collagenase II (2 mg/ml), BSA (2 mg/ml), DTT (1 mg/ml) in MEM with P/S for 1 h, centrifugation and mechanical dissociation. Tissue was then centrifuged (600 rpm, 5 min) and re-suspended in MEM with P/S. All diagnostic agents were purchased from Sigma-Aldrich, St. Louis, USA).

### Measurement of intracellular Ca^2+^ concentration in colon tissue and in single cells with calcium orange and fura-2

Colon strips with a length of 1 cm were removed and first cut longitudinally and then into smaller pieces. Tissue pieces were transferred onto cover slips and incubated individually for 5 min in MEM containing calcium orange (5 μM, Invitrogen) in 500 μl MEM.

For single cell experiments, cells were isolated like described above. Cells were loaded with calcium orange (5 μM) in 300 μl MEM. Stimulation was performed with denatonium (1 mM). Monitoring and analyses of changes in intracellular calcium concentration ([Ca^2+^]_i_) were performed with a confocal laser scanning microscope (LSM 710, Zeiss, Oberkochen, Germany) and Zenon software (Zeiss). Calcium orange was excited at 549 nm and fluorescence was recorded at 576 nm. Imaging speed was 1.57 frame/s. Before the experiments, brush cells were identified by their green fluorescence at 488 nm.

Isolated colonic cells including brush cells were seeded on cover slips and then loaded with fura-2 AM (5 μM) and sulfobromophthaleine (100 μM), a multi-drug resistance inhibitor, in 300μl MEM for 10 min at 37°C. Prior to measurement, cover slips with cells were transferred into a delta-T-dish fixed on a light microscope (Olympus BX50WI, Olympus, Hamburg, Germany) and constantly perfused (0.5 ml/min) with warmed MEM. The measurements were performed at constant perfusion at 30°C. Test stimuli were ATP (100 μM), cycloheximide (100 μM), and denatonium (1 mM). All substances were applied in a volume of 500 μl. Each cell was tracked independently and the fluorescence intensity ratio (340 to 380 nm) was calculated using a monochromator coupled to a slow-scan charged-coupled device camera system (Camera IMAGO, TILL Photonics, Gräfelfing, Germany) and analyzed with image analyses software (TILL Vision, TILL Photonics). Brush cells were detected by their fluorescence at 488 nm before the beginning of the experiments.

### Statistical analysis

For calcium imaging experiments, differences in signal intensities between the two cell types (EGFP-positive vs. EGFP-negative) were analyzed using unpaired *t*-test. A *p*-value < 0.05 was regarded a significant difference.

## Results

### Region-specific cholinergic phenotype of gastro-intestinal and biliary brush cells

**Figure 1:**
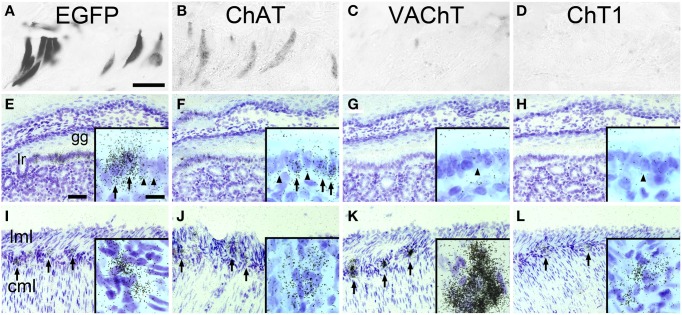
**The cholinergic phenotype of EGFP^*ChAT*^ brush cells in the mouse stomach**. **(A–D)** Brush cells in the mouse gastric groove-lining mucosal epithelium are immunoreactive for **(A)** EGFP^*ChAT*^ and **(B)** ChAT, but lack **(C)** VAChT and **(D)** ChT1 immunoreactivities. **(E–H)** *In situ* hybridization histochemistry (ISH) showing presence of **(E)** EGFP^*ChAT*^ and **(F)** ChAT transcripts, but absence of **(G)** VAChT and **(H)** ChT1 transcripts in tissue sections from ChAT-EGFP mice comprising the gastric groove. Insets show single cells with (arrow) and without (arrowhead) ISH signals. **(I–L)** ISH showing presence of **(I)** EGFP^*ChAT*^, **(J)** ChAT, **(K)** VAChT, and **(L)** ChT1 transcripts in neurons of the stomach myenteric plexus to prove suitability of the riboprobes used. Arrows point to selected myenteric ganglia, and examples of single cell expression shown in high magnification in insets. Note that all four riboprobes unequivocally detect cholinergic neurons in the myenteric plexus on the same respective section. Cml, circular muscle layer; gg, gastric groove; lr, limiting ridge; lml, longitudinal muscle layer. Bar in **(A)** equals 25 μm (for **A–D**). The bar in E equals 50 μm (for **E–L**). Bar in inset **(E)** equals 10 μm and applies to all insets.

ChAT-expressing cells have previously been detected is the mucosal epithelium lining the so-called “gastric groove” between fundus and corpus of the mouse stomach (Eberle et al., 2013b). Using an enzyme-linked immunohistochemical detection method, many cells in the gastric groove epithelium, also known as limiting ridge, stained EGFP^*ChAT*^ positive (Figure 1A). On adjacent sections, cells in the same region also stained positive for ChAT itself (Figure 1B), but lacked detectable presence of either VAChT (Figure 1C), or ChT1 (Figure 1D). On the transcript level, a high signal density for EGFP^*ChAT*^ mRNA was seen in many cells along the gastric groove epithelium by using *in situ* hybridization (Figure 1E). A similar pattern of ChAT-mRNA expressing cells was detected, although with lower signal strength than that for EGFP^*ChAT*^ (Figure 1F). Neither VAChT mRNA (Figure 1G), nor ChT1 mRNA (Figure 1H) was detectable in limiting ridge brush cells, while neurons in the myenteric plexus on the same section were strongly positive for all four riboprobes (Figures 1I–L, respectively).

**Figure 2:**
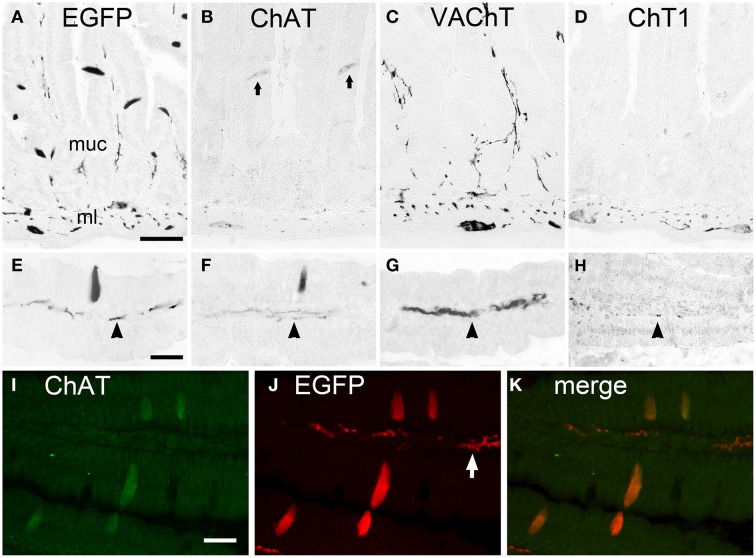
**The cholinergic phenotype of EGFP^*ChAT*^ brush cells in the mouse small intestine**. **(A–D)** In the duodenum, EGFP*^*ChAT*^* immunoreactivity depicts slender trans-epithelial cells in the mucosa (**A**, higher magn. in **E**), in addition to cholinergic nerve fibers (arrowheads in E-H) and neurons in the mucosal stroma and the muscle layer. Similarly, although with weaker staining intensity, ChAT immunoreactivity is detectable (**B+F**, arrows depict two stained epithelial cells). Both VAChT (**C+G**) and ChT1 (**D+H**) immunoreactivities are present in nerve fibers and neurons (note presence of ChT1 predominantly in ml), but absent from epithelial brush cells. **(I–K)** Double immunofluorescence for EGFP*^*ChAT*^* and ChAT showing full overlap in epithelial brush cells. Note preferential staining of subepithelial nerve fibers with the EGFP antibody compared to ChAT. ml, muscle layer; muc, mucosa. The bar in **(A)** equals 50 μm (for **A–D**), the bar in **(E)** equals 25 μm (for **E–H**), the bar in **(I)** equals 20 μm (for **I–K**).

The EGFP^*ChAT*^+/ChAT+/VAChT-/ChT1- phenotype of cholinergic brush cells was found to be identical in all segments of the small intestine. Shown here for the duodenum, EGFP*^*ChAT*^* immunoreactivity labeled solitary slender epithelial cells in the mucosa (Figures 2A,E), together with neuronal cell bodies and nerve fibers. ChAT immunoreactivity was present in these solitary epithelial brush cells and in intrinsic neurons and nerve fibers (Figures 2B,F). VAChT (Figures 2C,G) and ChT1 (Figures 2D,H) immunoreactivities labeled neuronal cell bodies in the myenteric plexus of the muscle layer, and nerve fibers running in the center of the mucosal stroma, but were completely absent from non-neuronal brush cells. Double immunofluorescence analysis proved that EGFP*^*ChAT*^*- and ChAT-positive brush cell staining fully overlapped (Figures 2I–K). This was also true for all other aspects of the gastro-intestinal and biliary systems analyzed (data not shown).

**Figure 3:**
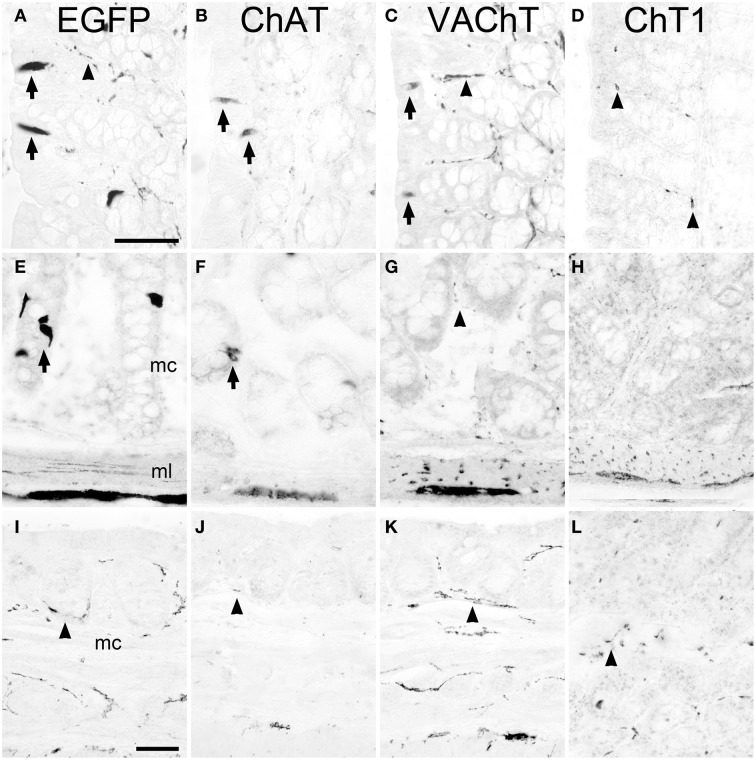
**The cholinergic phenotype of EGFP^*ChAT*^ brush cells in the mouse large intestine**. In the proximal colon, EGFP*^*ChAT*^* **(A)**, ChAT **(B)**, and VAChT **(C)** immunoreactivities are present in both epithelial cells (arrows) and in neurons and nerve fibers (arrowheads), while sparse ChT1 **(D)** labeling is restricted to nerve fibers (arrowheads). In medial and distal aspects of the colon, EGFP*^*ChAT*^* **(E)**, and ChAT **(F)** immunoreactivities show identical staining patterns (arrows point to epithelial brush cells), while both VAChT **(G)** and ChT1 **(H)** are restricted to nerve fibers and neurons (see arrowhead in **G**). **(I–L)** In the anal canal cholinergic brush cells are absent and immunolabeling is restricted to nerve fibers (arrowheads in **I–L**). mc, mucosal layer; ml, muscle layer. The bar in **(A)** equals 50 μm (for **A–H**), the bar in **(I)** equals 50 μm (for **I–L**).

A region-selective difference in the cholinergic phenotype of brush cells was noted in the proximal part of the large intestine, i.e., the ascending colon. Here, besides presence of EGFP^*ChAT*^ (Figure 3A) and ChAT (Figure 3B), VAChT-immunoreactivity was also detected in non-neuronal cells (Figure 3C). These VAChT-positive mucosal cells stained co-immunoreactive for EGFP, indicating that they were brush cells (data not shown) and represented about 20% of the total number of EGFP*^*ChAT*^*-immunoreactive cells. ChT1-immunoreactivity was again not detected (Figure 3D). The cholinergic phenotype of brush cells in the more distal parts of the large intestine was again like that of the small intestine, with presence of EGFP*^*ChAT*^* (Figure 3E) and ChAT (Figure 3F), and absence of VAChT (Figure 3G) and ChT1 (Figure 3H). In the anal canal, EGFP*^*ChAT*^* immunoreactive brush cells were practically absent (Figure 3I), and EGFP*^*ChAT*^*, ChAT (Figure 3J), VAChT (Figure 3K), and ChT1 (Figure 3L) immunoreactivities confined to nerve fibers. In contrast to the stomach, a limiting ridge-like region was not found to be established at the border between the mucosa of the anal canal and the surface epidermis (not shown).

**Figure 4:**
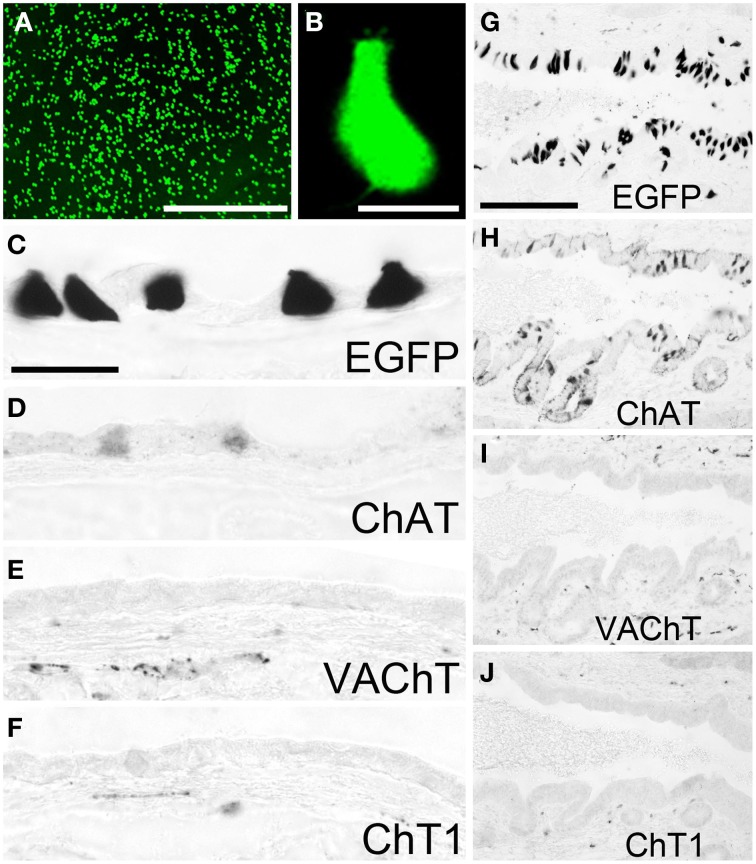
**The cholinergic phenotype of EGFP^*ChAT*^ brush cells in the mouse gall bladder and bile duct. (A)** Native EGFP^*ChAT*^ fluorescence from flat-mounted whole gall bladder. **(B)** High magnification of a single EGFP^*ChAT*^ fluorescent brush cell. Brush cells in gall bladder (**C–F**) and bile duct **(G–J)** are immunoreactive for EGFP (**C,G**) and ChAT (**D,H**), but lack detectable expression of VAChT (**E,I**) and ChT1 (**F,J**). Note that C and D are derived from non-adjacent sections and thus show different cell numbers. Also note subepithelial cholinergic nerve fibers detected with VAChT and ChT1 antibodies in the gall bladder and bile duct walls. The bar in **(A)** equals 200 μm. The bar in **(B)** equals 10 μm. The bar in **(C)** equals 25 μm and applies to **(C–F)**. The bar in **(G)** equals 100 μm and applies to **(G–J)**.

Non-neuronal cells expressing a ChAT phenotype have also been found abundant in the gall bladder (Höfer and Drenckhahn, 1998; Gautron et al., 2013). Using native tissue samples from the gall bladder of ChAT-EGFP mice, we observed numerous EGFP*^*ChAT*^*-fluorescent cells throughout the mucosa with highest concentration in the neck region (Figure 4A). Most of these cells had a pear-like shape and fine protrusions at their apical pole (Figure 4B). Immunohistochemical analysis identified these cells positive for EGFP*^*ChAT*^* (Figure 4C) and ChAT (Figure 4D), but lacking detectable amounts of both VAChT (Figure 4E), and ChT1 (Figure 4F). A similar high density of EGFP*^*ChAT*^*-immunoreactive cells was present all along the bile duct, shown here for the distal pancreatic part close to the entrance into the duodenum (Figure 4G). Again, these cells were also immunoreactive for ChAT (Figure 4H), but lacked detectable amounts of VAChT (Figure 4I) and ChT1 (Figure 4J).

### Cholinergic gastro-intestinal and biliary brush cells are separate from enteroendocrine cells

**Figure 5:**
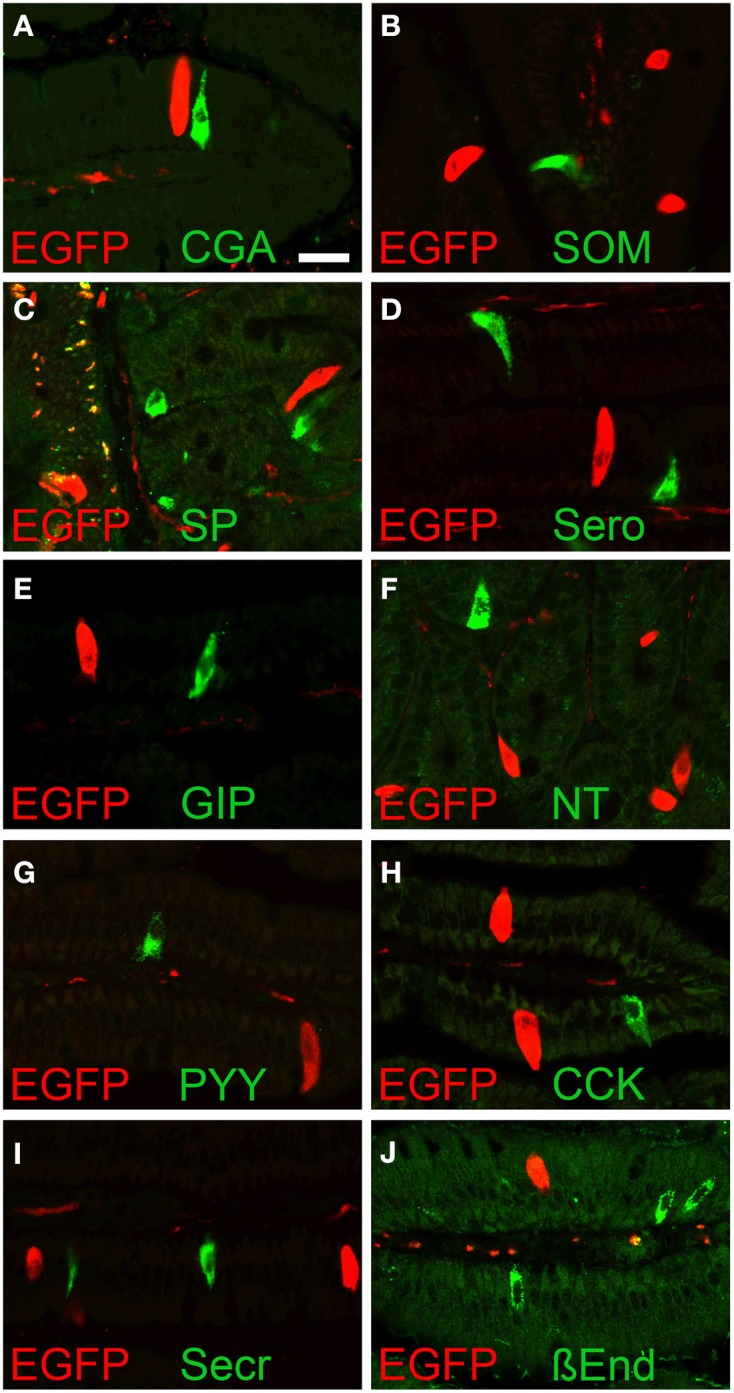
**EGFP*^*ChAT*^* brush cells and enteroendocrine cells are separate entities**. Double immunofluorescence analysis showing that EGFP*^*ChAT*^* immunoreactive brush cells in the duodenum do not co-localize with the enteroendocrine cell markers **(A)** chromogranin A (CgA), **(B)** somatostatin (SOM), **(C)** substance P (SP), **(D)** serotonin (Sero), **(E)** glucose-dependent insulinotropic peptide (GIP), **(F)** neurotensin (NT), **(G)** peptide tyrosin tyrosin (PYY), **(H)** cholecystokinin (CCK), **(I)** secretin (Secr), and **(J)** ß-endorphin (ß End). The bar in **(A)** equals 20 μm and accounts for all pictures.

To evaluate if the EGFP*^*ChAT*^* immunoreactive cells were enteroendocrine cells, we performed double immunohistochemical detection of EGFP*^*ChAT*^* with chromogranin A (CgA), a marker for many enteroendocrine cell types (Weihe et al., 1994). Shown here for the duodenum (Figure 5A), EGFP*^*ChAT*^* immunoreactive brush cells were separate from CgA-immunoreactive enteroendocrine cells (*n* > 500 cells from four mice investigated). Strict segregations of EGFP*^*ChAT*^* immunoreactive brush cells and CgA-positive enteroendocrine cells were also seen in stomach, jejunum, ileum, and colon, while gall bladder and bile duct did not contain CgA-positive cells. In addition, a comparison of EGFP*^*ChAT*^* immunoreactive cells with markers for intestinal enteroendocrine cell subtypes revealed strict segregation in all aspects of the gastro-intestinal tract. Exemplified for the duodenum, somatostatin-expressing D-cells (Figure 5B, *n* > 200 cells investigated), substance P-positive SubP-cells (Figure 5C, *n* > 250), serotonin-positive EC-cells (Figure 5D, *n* > 600), glucose-dependent insulinotropic peptide-positive K-cells (Figure 5E, *n* > 500), neurotensin-positive N-cells (Figure 5F, *n* > 20), peptide YY-positive L-cells (Figure 5G, *n* > 50), cholecystokinin-positive I-cells (Figure 5H, *n* > 400), secretin-positive S-cells (Figure 5I, *n* > 600), and ß-endorphin-positive cells (Figure 5J, *n* > 50) all were found non-overlapping with the EGFP*^*ChAT*^* cells.

### Cholinergic gastro-intestinal and biliary brush cells express CK18 and TRPM5

**Figure 6:**
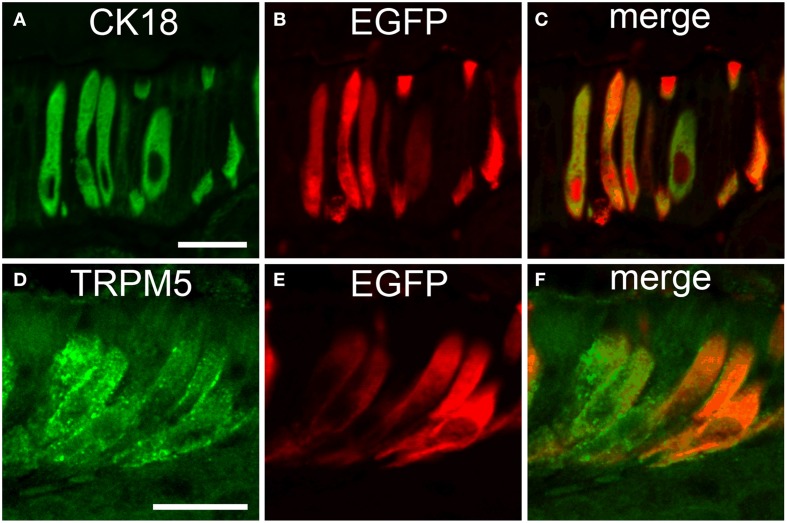
**EGFP^*ChAT*^ brush cells express CK18 and TRPM5**. Double immunofluorescence analysis shows that all EGFP*^*ChAT*^* immunoreactive cells in the gastric groove epithelium are co-positive for CK18 **(A–C)** and TRPM5 **(D–F)**. The bars in **(A)** and **(C)** equal 20 μm.

To further evaluate if the non-neuronal EGFP*^*ChAT*^* cells were identical with the previously characterized taste-perceiving like cell population, we performed double-immunofluorescence analysis for EGFP*^*ChAT*^* with the brush cell marker CK18 and for TRPM5. CK18 and EGFP*^*ChAT*^* immunoreactivities fully overlapped in the epithelium at the gastric groove (Figures 6A–C, *n* > 100) and in the gall bladder wall (*n* > 120, data not shown). Cells with single staining for either one of the two markers were absent. While in the small intestine a large number of epithelial cells stained for CK18 with varying intensity, this was true for all epithelial cells in the colon. EGFP*^*ChAT*^* always co-localized to the fraction of strongly positive CK18-expressing cells (< 10% in small intestine, *n* > 200; <<1% in colon, *n* > 200) in both locations (data not shown). In addition, EGFP*^*ChAT*^* cells displayed full overlap with TRPM5 in all areas analyzed (*n* > 500), shown here for the stomach (Figures 6D–F).

### Cholinergic gastro-intestinal brush cells are capable to synthesize prostaglandin-D2

**Figure 7:**
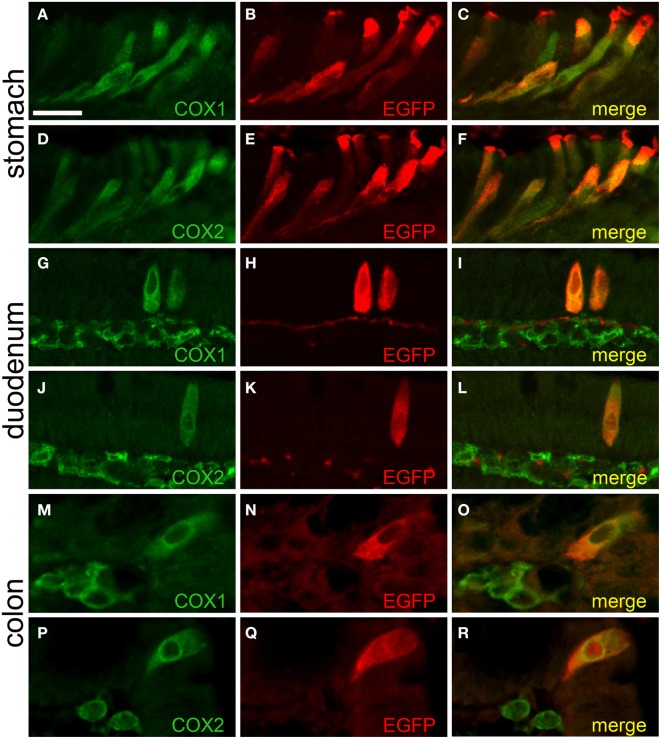
**EGFP^*ChAT*^ brush cells express COX1 and COX2**. Double immunofluorescence analyses showing that EGFP*^*ChAT*^* immunoreactive brush cells in stomach **(A–F)**, duodenum **(G–L)**, and colon **(M–R)** are co-positive for COX1 **(A,G,M)**, and COX2 **(D,J,P)**. Note that in the intestine also cells in the subepithelial tissue stain positive for COX1 and COX2, but not for EGFP. The bar in **(A)** equals 20 μm and accounts for all panels.

We found that the EGFP*^*ChAT*^*-immunoreactive brush cells selectively express the key enzymes for prostaglandin-D2 synthesis, cyclooxygenases 1 (COX1) and 2 (COX2) (also referred to as prostaglandin-endoperoxide synthases, Ptgs1 and Ptgs2), as seen before by others co-staining with TRPM5 (Bezençon et al., 2008; Eberle et al., 2013a). Shown for stomach (Figures 7A–F), duodenum (Figures 7G–L), and colon (Figures 7M–R), EGFP*^*ChAT*^* immunoreactivity fully overlapped with both COX1 and COX2.

### EGFP^*ChAT*^ brush cells of the mouse colon respond to the bitter stimulus, denatonium

**Figure 8:**
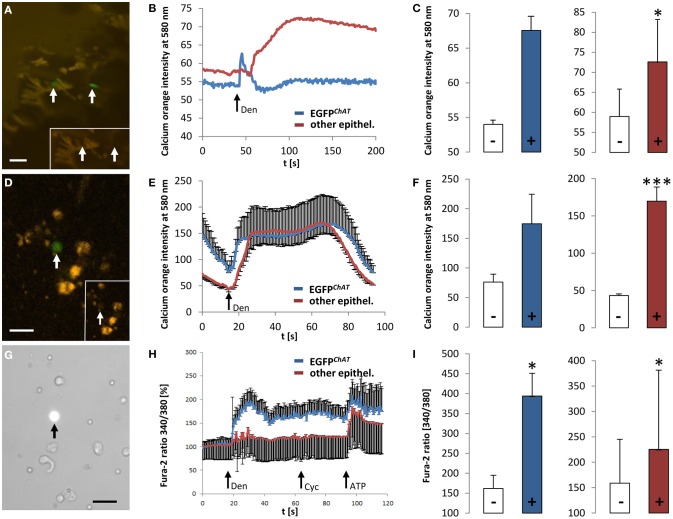
**Responsiveness of colonic EGFP^*ChAT*^ brush cells in comparison to other epithelial cells to bitter stimuli. (A)** Loading of colon epithelial cells with calcium orange in a colon tissue preparation. Arrows point to EGFP*^*ChAT*^* fluorescent cells. The inset depicts low loading efficiency of two EGFP*^*ChAT*^* cells compared to surrounding other epithelial cells. **(B)** Change in calcium orange signal intensity after stimulation with denatonium (arrow marks stimulus initiation). Note delay in signal rise and signal maximum in other epithelial cell compared to EGFP*^*ChAT*^*. **(C)** Comparison of calcium orange intensity changes before (−) and after (+) stimulation with denatonium for EGFP*^*ChAT*^* (blue bar) and other epithelial (red bar) cells. **(D)** Loading of dissociated colon epithelial cells with calcium orange. The arrow points to a EGFP*^*ChAT*^* fluorescent cell. The inset depicts low loading efficiency of this EGFP*^*ChAT*^* cell compared to surrounding other epithelial cells. **(E)** Change in calcium orange signal intensity after stimulation with denatonium. Note delay in reaching signal maximum in other epithelial cell compared to EGFP*^*ChAT*^*. **(F)** Comparison of calcium orange intensity changes before (−) and after (+) stimulation with denatonium for EGFP*^*ChAT*^* (blue bar) and other epithelial (red bar) cells. **(G)** Loading of dissociated colon epithelial cells with fura-2. The arrow points to a EGFP*^*ChAT*^* fluorescent cell. **(H)** Change in fura-2 ratio [%] after stimulation with denatonium (Den), cycloheximide (Cyc), and ATP. Data was normalized to 100% before stimulation. Note that the delayed increase in other epithelial cells did not occur at the same time in every cell analyzed. Thus, the average response shows several peaks. **(I)** Comparison of fura-2 ratio before (−) and after (+) stimulation with denatonium for EGFP*^*ChAT*^* (blue bar) and other epithelial (red bar) cells. Data represents absolut values. ^*^p < 0.05; ^***^p < 0.001 (*t*-test). The size bars in **(A,D,G)** are 20 μm.

Co-localization of TRPM5 and EGFP*^*ChAT*^* in brush cells (see above) is in line with our previous idea that brush cells may have chemosensory functions (Kaske et al., 2007; Finger and Kinnamon, 2011). To further test this concept we performed *ex vivo* Ca^2+^ imaging experiments on colon tissue preparations and on isolated cells, all taken from the ChAT-EGFP mice. First, we measured changes in [Ca^2+^]_i_ evoked by the typical bitter taste stimulus, denatonium. After loading colon tissue preparations with the calcium-sensitive dye, calcium orange, it became obvious that the loading efficiency of EGFP*^*ChAT*^*-positive brush cells was poor when compared to other epithelial cells (Figure 8A). However, the application of denatonium to the bath solution elicited a fast peak in a few EGFP*^*ChAT*^*-positive brush cells (*n* = 2 cells from one animal), while a delayed increase in signal was observed in non-brush cells (*n* = 8/1) (Figure 8B). In both cell populations the calcium orange signal was raised to a similar extent (EGFP*^*ChAT*^*-positive cells: from 54 ± 0.9 to 67 ± 2.65, Δ 13.45 ± 4.78; other epithelial cells: from 58.9 ± 7.88 to 72.6 ± 10.8, Δ 13.64 ± 3.35, *p* < 0.05) (Figure 8C). When using dissociated colon cells instead, the cell loading with calcium orange was again much better in other epithelial cells compared to EGFP*^*ChAT*^*-positive brush cells (Figure 8D). As seen before, both cell populations showed increases in the calcium orange signal after stimulation with denatonium (Figure 8E). Again, non-brush cells showed a delayed rise in [Ca^2+^]_i_, and both cell populations reacted with a similar intensity (EGFP*^*ChAT*^*-positive brush cells: from 76.06 ± 13.34 to 174.53 ± 54.31, Δ 98.46 ± 16.37, *n* = 3/3; other epithelial cells: from 43.24 ± 4.85 to 169.78 ± 21.99, Δ 126.54 ± 38.29, *p* < 0.001, *n* = 9/3) (Figure 8F).

We speculated that the inefficiency in loading EGFP*^*ChAT*^*-positive brush cells with dye compared to other epithelial cells was due to an active outward flow that led to a rapid elimination of the dye substances. The loading of EGFP*^*ChAT*^*-positive brush cells with fura-2 in combination with sulfobromophthaleine was much more efficient (Figure 8G). After application of denatonium, EGFP*^*ChAT*^*-positive brush cells showed a rapid and strong increase in fura-2 signal (from 162.43 ± 33.87 to 394.21 ± 57.34, Δ91.59 ± 21.6, *p* < 0.05, *n* = 9/2), while other epithelial cells displayed a delayed and significantly weaker increase (from 158.73 ± 86.43 to 225.31 ± 156.12, Δ 24.33 ± 13.7, *p* < 0.05, *n* = 62/2; *p* < 0.01 between the two cell populations) (Figures 8H,I). This delayed increase in other epithelial cells did not occur at the same time in every cell analyzed. Thus, the average response showed several peaks (Figure 8H). Interestingly, both cell populations did not react upon stimulation with cycloheximide in the same experiment (EGFP*^*ChAT*^*-positive brush cells: from 364.98 ± 43.6 to 389.3 ± 36.45; other epithelial cells: from 225.31 ± 156.12 to 241.77 ± 158.41, *p* = n.s.). Finally, the response to the control stimulus ATP was comparably strong (EGFP*^*ChAT*^*-positive brush cells: from 367.8 ± 46.8 to 504.73 ± 58.5, *p* < 0.05; other epithelial cells: from 225.06 ± 143.47 to 373.24 ± 320.21, *p* < 0.05; *p* = n.s. between the two cell populations), proving (i) the viability of the investigated cells until the end of the experiments, (ii) their ability to respond multiple times to stimuli that raise [Ca^2+^]_i_, and (iii) a comparable loading with fura-2 of the investigated cell populations.

## Discussion

The concept of taste perception in the gut mucosa and adnexa by brush cells has been put forward more than two decades ago. However, molecular and mechanistic insight into how brush cells develop, how they sense the luminal content, which substances they perceive, and how they transmit this information in a paracrine fashion to neighboring cells or nerve fibers is up to date known only in fragments (Gerbe et al., 2012). The recent identification of ACh production in brush cells of many body inner surfaces, e.g. nose, auditory tube, trachea, gut, and urethra, due to unequivocal detection of ChAT expression, has boosted descriptive and mechanistic studies centering on the role of non-neuronally produced ACh. In the present study, by utilizing EGFP*^*ChAT*^* transgenic mice, we show that most ChAT-expressing brush cells along the GI and biliary tracts: (1) express an incomplete neuronal-type cholinergic phenotype, with presence of ChAT but lack of detectable VAChT and ChT1, (2) are separate from the enteroendocrine cell lineage, (3) express a downstream component of the canonical taste transduction cascade, i.e., TRPM5, (4) express COX1 and COX2, and (5) are capable to detect the bitter substance denatonium, and respond with rises in intracellular calcium.

Evidence for a cholinergic nature of brush cells in certain parts of the GI and biliary tract has been provided recently (Eberle et al., 2013b; Gautron et al., 2013). According to our study the mouse anal canal, like esophagus, is a part of the GI tract devoid of cholinergic brush cells, and a band-like arrangement of brush cells comparable to that of the stomach was absent from the transition zone between the mucosa of the anal canal and the epidermis. The existence of brush cells in the mouse gall bladder epithelium has been known for almost 50 years (Luciano and Reale, 1969), however this cell did not receive much attention up to date, except on the ultrastructural level (Luciano and Reale, 1990). More recently, brush cells were described numerous in the rat pancreatic (Höfer and Drenckhahn, 1998) and bile ducts (Iseki, 1991). We here provide additional evidence that all gall bladder and biliary tract brush cells have a cholinergic, ChAT phenotype. Since this cell type makes up about 30% of all epithelial cells in these compartments (Iseki, 1991), we speculate that it serves a prominent role e.g., in preventing ascending bacterial infections.

In fully functional cholinergic neurons, expression of ChAT, VAChT, and ChT1 is obligate, but transcription ratios between ChAT and VAChT from the CGL can differ dramatically (Schütz et al., 2001). Their concomitant expression in non-neuronal brush cells has been shown for trachea (Krasteva et al., 2011), auditory tube (Krasteva et al., 2012b), and type II taste cells in lingual taste buds (Ogura et al., 2007). All other sites where non-neuronal cholinergic cells have been described rely solely on the detection of ChAT and its product, ACh. While the complete dissociation of expression of the two products of the CGL in non-neuronal locations by its own is surprising, our lack of detection of VAChT expression in most brush cells in the GI and biliary tracts implies that ACh is not released via small synaptic vesicles from these cells. The presence of some synaptic vesicle-associated proteins in gastric and intestinal brush cells has been documented (Bezençon et al., 2008; Eberle et al., 2013b), and the synaptic release of taurine from intestinal brush cells hypothesized (Bezençon et al., 2008). However, since VAChT is absent from most cholinergic brush cells, ACh shall be released in a different mode, possibly via plasma-membrane-bound polyspecific organic cation transporters (Wessler et al., 2001; Kummer et al., 2008; Pochini et al., 2012), the proteolipid *mediatophore* (Bloc et al., 1999), or maybe even gap junction hemichannels (Huang and Roper, 2010). Furthermore, absence of secretory granules from intestinal brush cells is supportive of a non-vesicle operated ACh release mode (Iseki and Kondo, 1990). Finally, in central and peripheral neurons, expression from a transgenic CGL led to a 5–8 fold transcriptional up-regulation of the embedded VAChT (Schütz et al., 2008). Thus, if VAChT were to be expressed also in all GI cholinergic brush cells in our EGFP*^*ChAT*^* mouse model this should have been reliably detectable. Our detection of VAChT immunoreactivity selectively in some EGFP-positive brush cells of the proximal colon suggests that sub-classes of cholinergic brush cells with different functions exist along the mouse GI tract. If these colonic VAChT-containing brush cells are functionally more related to their counterparts in the airways than to non-VAChT expressing brush cells in the other parts of the GI and biliary tracts needs to be addressed in subsequent studies.

We did not detect ChT1 expression in brush cells using *in situ* hybridization and immunohistochemistry, although its presence in GI and biliary tract nervous system structures was unequivocal. This contrasts data obtained from rat trachea (Pfeil et al., 2003) and colon (Bader et al., 2014). A re-uptake of choline by organic cation transporters in mice, like proposed for the rat (Bader et al., 2014), or through choline transporter-like proteins (Song et al., 2013) should be taken into consideration.

Phenotypic characterization of brush cells in the mouse GI tract has revealed that they express components of the canonical taste transduction cascade, i.e., α-gustducin and TRPM5 (Iwatsuki and Uneyama, 2012). We show that all investigated EGFP*^*ChAT*^* expressing cells are immunoreactive for TRPM5, but completely separate from enteroendocrine cells, at least of those specific sub-phenotypes tested here. Our data thus lend support to the concept that cholinergic brush cells in the GI and biliary tracts play a role in chemosensation, but not in peptide hormone actions. Other non-cholinergic, TRPM5-positive brush cells thus must exist that fulfill these other tasks (Kokrashvili et al., 2009).

We demonstrate for the first time the responsiveness of cholinergic brush cells from mouse colon *ex vivo* in both a more “organotypic” surrounding, i.e., in a colon tissue preparation, and in dissociated cells. An increase in [Ca^2+^]_i_ in response to stimulation with denatonium was detected in both cell populations. However, appropriate loading of isolated cells resulted in a superior change in fura-2 signal upon denatonium stimulation in EGFP*^*ChAT*^*-positive cells when compared to other epithelial cells, while the latter did not perform better and thus had elicited their maximum response independent of the addition of a multi-drug resistance inhibitor. Noteworthy, non-EGFP cells seemed to react with a timely delay when compared to EGFP*^*ChAT*^*-positive cells. Since the time-delayed response of non-brush cells was observed in all our experimental setups we speculate that these cells react to ACh released by the cholinergic brush cells, and not to the primary bitter stimulus. Further experiments beyond the scope of our current report are required to address this important issue.

Both cell population tested in our *ex vivo* experiments did not respond to cycloheximide, another bitter taste stimuli. Absence of expression of Tas2R105 in colon epithelial cells, or downregulation of receptor gene expression during the process of cell isolation may account for this property.

The capability of chemosensory (cholinergic) brush cells to sense taste-like substances has previously been studied in individual brush cells detached from their natural surroundings, i.e., from trachea (Krasteva et al., 2011), urethra (Deckmann et al., 2014), and nasal epithelium (Gulbransen et al., 2008). These studies have shown that brush cells are polymodal sensors for bitter and umami taste, as well as bacterial products, i.e., *quorum sensing molecules* (Tizzano et al., 2010; Krasteva et al., 2012a; Saunders et al., 2014). Our findings add to this concept the cholinergic gastro-intestinal brush cell as specific sensors for bitter-tasting substances from the gut lumen.

Since the whole gut is colonized by diverse types of “good” bacteria that help to digest food before uptake into enterocytes one can speculate that cholinergic brush cells serve a prominent sentinel function in detecting potentially harmful, bitter substances produced by these bacteria, and subsequently eliciting ACh-driven responses to eliminate the thread and prevent e.g., the bile duct, gall bladder, and liver from an ascending bacterial infection. Another role for these cells in gastrointestinal chemosensation could lie in monitoring the progress of fermentation along the gut to initiate and regulate intestinal responses, e.g., motility and bicarbonate secretion.

Other potentially paracrine signaling molecules specifically expressed by cholinergic brush cells are prostaglandins. We show that expression of both COX1 and COX2 fully overlaps with EGFP*^*ChAT*^* in GI and biliary tract epithelia, which was also described recently for TRPM5-positive brush cells of stomach (Eberle et al., 2013a) and intestine (Bezençon et al., 2008). This suggests that prostaglandins may be released from the cholinergic brush cells in addition to ACh and act as local chemical messengers to modulate inflammatory reactions, e.g., in ulcerative colitis (Jönsson et al., 2007).

In conclusion, our experiments revealed that cholinergic brush cells in the intestinal epithelium are sensors for the bitter stimulus denatonium when this is present on the luminal side. The restriction of ChAT and TRPM5 expression to a minor subpopulation of epithelial cells renders the cholinergic brush cell chemically uniquely equipped to serve a putative chemosensory function distinct from enterocytes or other epithelial cells, e.g., goblet or enteroendocrine cells. Further mechanistic studies comparable to those performed recently for trachea (Krasteva et al., 2011), nose (Saunders et al., 2014), and urethra (Deckmann et al., 2014) need to address the questions whether these cells really use ACh and prostaglandins as paracrine signaling molecules, on which local cells or nerve fibers these signaling molecules act, and which functional importance this has in the different aspects of the gastro-intestinal tract on maintaining e.g., homeostasis of intestinal epithelial cell growth and differentiation (Takahashi et al., 2014), or host defense scenarios against harmful substances and bacterial pathogens.

### Conflict of interest statement

The authors declare that the research was conducted in the absence of any commercial or financial relationships that could be construed as a potential conflict of interest.

## Abbreviations

ACh: acetylcholine
CgA: chromogranin A
CGL: cholinergic gene locus
ChAT: choline acetyltransferase
ChT1: high-affinity choline transporter 1
COX: cyclooxygenase
EGFP: enhanced green fluorescent protein
GI: gastro-intestinal
TRPM5: transient receptor potential melastatin-like subtype 5 channel
VAChT: vesicular acetylcholine transporter.
